# The Role of the Immune Cells in Fracture Healing

**DOI:** 10.1007/s11914-018-0423-2

**Published:** 2018-03-05

**Authors:** Gurpreet S. Baht, Linda Vi, Benjamin A. Alman

**Affiliations:** 1 Department of Orthopaedic Surgery, Duke Molecular Physiology Institute, Duke University, DUMC 104775, 300 North Duke Street, Durham, NC 27701 USA; 2 Duke Molecular Physiology Institute, Durham, NC USA; 3 Department of Orthopaedic Surgery, Duke University, 200 Trent Drive, Orange Zone 5th floor, Durham, NC 27710 USA; 4 University of Toronto, Toronto, Canada

## Abstract

**Purpose of review:**

Bone fracture healing is a complex physiological process relying on numerous cell types and signals. Inflammatory factors secreted by immune cells help to control recruitment, proliferation, differentiation, and activation of hematopoietic and mesenchymal cells. Within this review we will discuss the functional role of immune cells as it pertains to bone fracture healing. In doing so, we will outline the cytokines secreted and their effects within the healing fracture callus.

**Recent findings:**

Macrophages have been found to play an important role in fracture healing. These immune cells signal to other cells of the fracture callus, modulating bone healing.

**Summary:**

Cytokines and cellular signals within fracture healing continue to be studied. The findings from this work have helped to reinforce the importance of osteoimmunity in bone fracture healing. Owing to these efforts, immunomodulation is emerging as a potential therapeutic target to improve bone fracture healing.

## Introduction

In the context of tissue repair, bone is unique as it is able to heal itself without forming a scar. The lifetime prevalence of bone fracture is 50% in the US and while most bone injuries are able to heal normally, 5%–10% result in non-union every year. This rate increases with certain comorbidities and with advanced age [1, 2]. Approximately 100,000 fractures require surgical intervention every year in the US, amounting to over a billion dollars in health care costs [3]. Treatments thus far involve the use of frames to stabilize bone and of osteo-inductive agents (such as BMP) to increase the amount of bone deposition at the site of injury.

**Figure 1.**
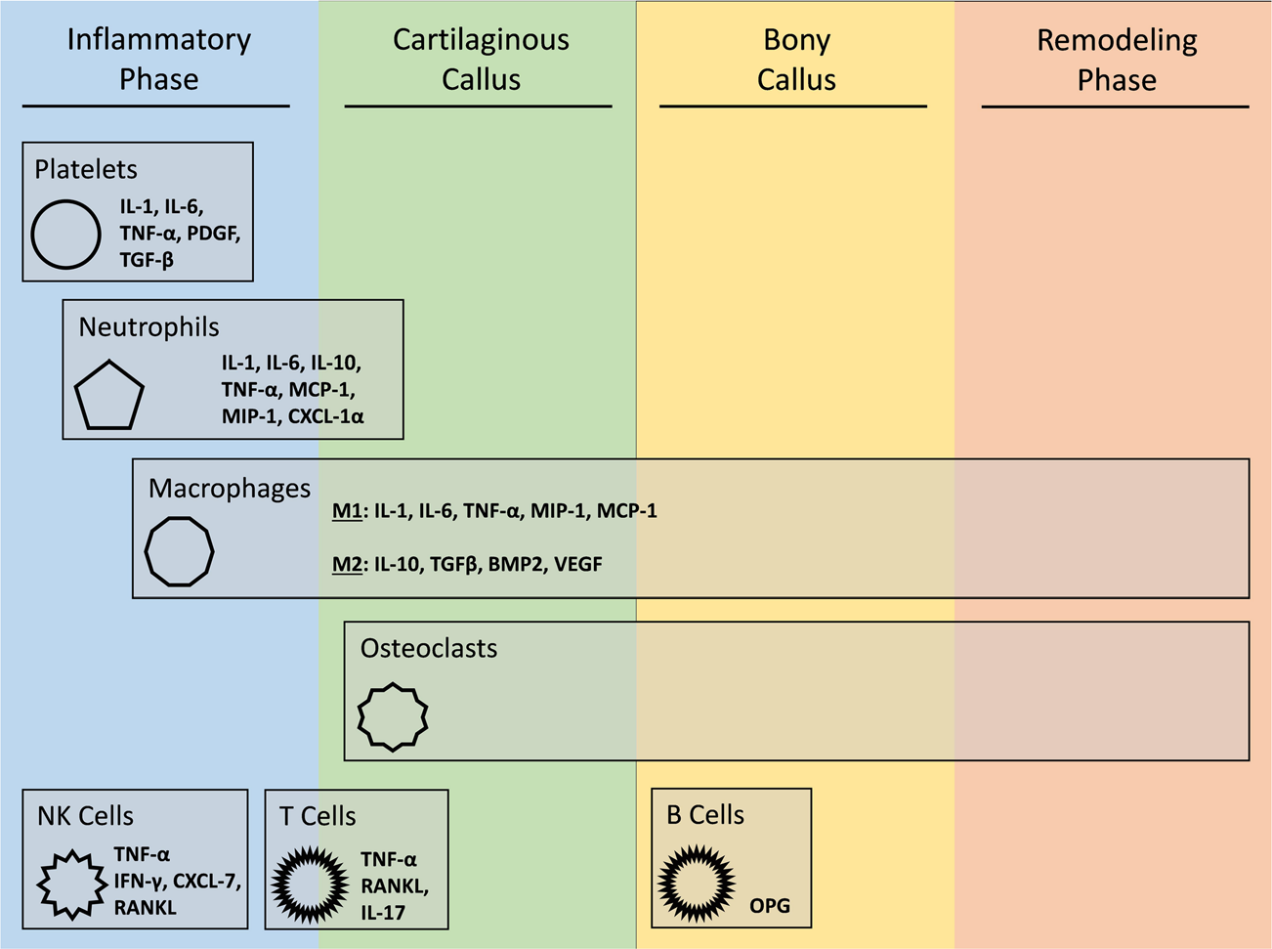
Role of immune cells during fracture repair. Bone fracture healing can be viewed as a four-stage process. Immune cells play important roles throughout this process; however, a majority of their activity occurs during early stages of fracture healing.

Recent work highlighting the importance of immune cells in fracture healing may develop into a potentially new area of treatment for bone injury [5–7]. Immune cells, which are derived from hematopoietic stem cells, are required for normal bone development and proper fracture healing. Dysfunction of these cells, as seen with age or with metabolic dysregulation, hinders bone repair. Rejuvenation of the hematopoietic population through parabiosis or bone marrow transplantation is able to ameliorate these shortcomings [4, 5]. Current efforts are directed at delineating the exact role these cells play in fracture healing. A better understanding of osteoimmunity could result in the emergence of therapeutic targets for bone healing.

When a bone is injured, an inflammatory response is cast: immune cells are recruited to the site of injury; multiple factors are secreted, inflammatory and other. This intense inflammatory event is required to ensure normal fracture healing through angiogenesis of vessels, repair of the injured tissue, and eventually remodeling [6–8]. While the inflammatory response itself is short-lived, the effects of the immune cells extend beyond the early stages of fracture healing. Mesenchymal progenitor cell recruitment and activation rely on this inflammatory response. Thus, immune cells are integral to bone fracture healing. This review will outline the role of the immune system during fracture healing, then specifically the role of each individual cell type.

## Bone Fracture Healing

Fracture repair is a complex and well-orchestrated regenerative process involving numerous signaling pathways and cell types. It follows one of two processes: (1) primary (direct) fracture repair, and (2) secondary fracture repair.

Primary fracture repair involves deposition of bone tissue: mesenchymal progenitor cells and osteoblasts are recruited and activated at the site of injury; subsequently, they deposit a bone matrix to unify the tissue [9]. This type of fracture healing occurs when the fractured bone-ends are rigidly fixed and lack relative displacement, leading to little or no inflammatory response. While this process is not likely to occur spontaneously in nature, it does ensue in fracture following fixation (such as in distraction osteogenesis in limb lengthening) [10].

Secondary fracture repair is the more common form of fracture healing observed in clinic. Bone repair occurs through a cartilaginous intermediate and in phases described below. Immune cells play a significant role in secondary bone repair.

### Inflammatory Phase

Long-bone fracture healing is likened to endochondral ossification seen during embryonic development, but a key difference is the presence of an inflammatory phase during the former. When a fracture occurs, there is a local disruption of the vascularization and soft tissue. In response to this vascular injury, a hematoma forms and will act as the future template for callus formation [11]. Immune cells, including platelets, neutrophils, and macrophages are then recruited to the site and activated [12, 13]. These cells invade the hematoma and secrete growth factors and cytokines, which help to recruit mesenchymal cells. The hematoma is reorganized and there is deposition of a fibrin thrombus [11, 12]. As capillaries invade the thrombus, granulation tissue replaces the fibrin clot. Neutrophils and macrophages remove dead cells and debris [14]. They also release factors that promote the recruitment of mesenchymal progenitor cells that originate from the periosteum, bone marrow, and systemic circulation [15, 16]. These cells, in turn, have an immunosuppressive character which helps to resolve the inflammation at the site and prepare it for the next stage of healing [17–21].

### Cartilaginous Callus Formation

During the inflammatory phase, mesenchymal progenitor cells are recruited to the site of injury and undergo chondrogenic differentiation. It is the decreased mechanical stability at the fracture site that promotes this chondrogenesis. The granulation tissue is replaced by a fibrocatilaginous callus which poses a semi-rigid quality to provide mechanical support [8, 11]. Initially, this cartilaginous callus is largely avascular; however, as healing proceeds, the callus is invaded by endothelial cells, promoting angiogenesis [22]. This induces terminal differentiation of chondrocytes, resulting in hypertrophy and the production of mineralized cartilaginous matrix [8, 23]. The fate of these chondrocytes is now debated as either undergoing apoptosis or undergoing trans-differentiation/dedifferentiation to osteogenic cells [24].

### Bony Callus Formation

Upon calcification of the fracture callus, osteoprogenitor cells are recruited from the periosteum, bone marrow, vasculature, and surrounding tissue to initiate osteogenesis and the deposition of bone onto the calcified cartilage [8]. Simultaneously, osteoclasts are activated at the site and begin to resorb the cartilaginous callus. This results in the replacement of the cartilaginous callus with the bony callus, which is composed of woven bone and provides greater stability than the fibrocartilaginous callus [25]. Macrophages and T and B cells have been shown to play a role during mineralization (discussed below); however, their function has yet to be elucidated.

### Remodeling Phase

The remodeling phase marks the last stage of fracture repair. Woven bone within the callus is replaced with laminar bone, consisting of a highly organized matrix of collagen fibers; therefore restoring the original structure and function of the bone [11]. This process is driven by osteoclast-­-mediated bone resorption followed by osteoblast-­-mediated bone formation [8].

### Immune Cells and Fracture Healing

As described, fracture repair is a complex process requiring a well-organized response from multiple cell-types. During the inflammatory phase; clot formation, tissue granulation, and cell recruitment are necessary first steps which are dependent on the coordination of various immune cells. Throughout the different phases, hematopoietic cells appear to direct mesenchymal cell differentiation and activity. New information is rapidly being uncovered as osteoimmunity is a developing field. The following is a summary of immune cell types and their observed roles in fracture healing.

### Immune cell function and origin

Hematopoietic cells arise from the mesoderm during embryonic development and locate to numerous sites in the human body. Along with the spleen, the bone marrow serves as a primary source for hematopoietic cells during adulthood: from within it all cells of the hematopoietic lineage can be differentiated. These cells play important roles in staving off infection and identifying foreign bodies; they are divided into two groups: cells of the lymphoid lineage and cells of the myeloid lineage. The majority of the cells located within the bone marrow cavity remain in a quiescent, multipotent state and are activated upon stimulus. Bone injury often results in bleeding; damage to the local vasculature serves as an activation step to recruit and activate these cells.

### Platelets

Platelets are non-nucleated cells of the myeloid lineage. Their primary function lies in blood clotting; however, they have been shown to have a role in fracture healing [26]. Soon after injury, circulating platelets arrive at the affected site and are activated by the thrombin released in response to injured vasculature. Activated platelets take part in creating the fibrin thrombus. This acts as a scaffold for cellular engraftment as platelets secrete inflammatory cytokines (IL-1, IL-6, TNF-α) and growth factors (PDGF, TGF-beta) to recruit other immune cells (neutrophils and monocytes) and mesenchymal progenitor cells respectively [27–29].

### Neutrophils

Neutrophils are phagocytic cells of the myeloid lineage. As described, neutrophils are recruited by IL-1, TNF-α secreted by platelets [30, 31]. The role of neutrophils in fracture healing is still being elucidated and involves many aspects of tissue repair. During early stages of the inflammatory phase, neutrophils have been shown to contribute to the fibrin thrombus by depositing a fibronectin matrix [32]. During the later stages of the inflammatory phase, neutrophils take part in removing cellular and tissue debris and are implicated in removal of the thrombus [33–35]. However, their most significant role seems to involve the secretion of cytokines (IL-1, IL-6, IL-10, TNF-α, MCP-1, CXCL-1α, MIP-1) to attract monocytes, which will differentiate to macrophages [36–39].

### Macrophages

Macrophages are phagocytic cells of the myeloid lineage. They are differentiated from monocytes. These cells play an integral part in bone homeostasis as well as bone fracture repair. During homeostasis, macrophages likely act as niche cells to osteoblasts and to osteoclasts, taking part in the crosstalk and communication to maintain the balance in bone remodeling. Indeed, our work shows that ablation of macrophages retards early skeletal growth and development resulting in decreased trabecular number and decreased bone mineral density and later leads to osteoporosis [40•].

The importance of macrophages in fracture healing is still being investigated. The depletion of macrophages during bone fracture healing delays bone union [5, 40•, 41]. Fracture calluses from mice in which macrophages had been ablated developed smaller, under-mineralized fracture calluses with increased amounts of fibrotic tissue. Furthermore, depletion of macrophages decreased the number of mesenchymal progenitor cells and inhibited the ability of these cells to differentiate to osteoblasts [40•, 42••].

In fracture healing, monocytes are recruited to the site of injury by MIP-1 (also known as CXCL2) primarily, and by IL-1 and TNF-α. They subsequently differentiate to macrophages that have a sliding scale of functional attributes dependent on their “polarization”, which is induced by extracellular signals and is thought to be reversible in vivo [43]. At one end, macrophages undergo programming to become classically activated M1 macrophages when exposed to inflammatory cytokines (IL-1, TNF-α). These are inflammatory macrophages that further secrete IL-1, IL-6, TNF-α, MCP-1, and MIP-1 to maintain recruitment of monocytes. They perform phagocytosis to remove necrotic cells as well as the fibrin thrombus [45]. At the other end of the sliding scale are the alternatively activated M2 macrophages, which are functional after exposure to IL-4. These cells initiate an anti-inflammatory response in the later stages of inflammation as they secrete tissue repair signals (IL-10, TGF-beta, BMP-2, and VEGF), recruit mesenchymal progenitor cells, induce osteochondral differentiation, and prompt angiogenesis [46–50].

The importance of macrophages in tissue homeostasis has be confirmed in other tissues as well [44–47]. Interestingly, tissue-resident macrophages have been found to be of benefit to tissue health while macrophages derived from circulating monocyte have been found to be less efficient [48]. Recently, the existence of tissue resident macrophages in bone (termed Osteomacs) has been proposed [42••, 49]. The comparative importance of tissue-resident versus monocyte-driven macrophages in bone biology has yet to be elucidated.

### Osteoclasts

Osteoclasts are multinucleated cells of the myeloid lineage; they differentiate directly from monocytes, although they can also arise from macrophages [50].

Although osteoclasts are not traditionally thought of as immune cells, they are able to act as innate immune cells within bone as inflammatory signals lead to differentiation and activation of osteoclasts [51].

Osteoclasts are specialized cells as they are the only cells that resorb bone matrix [52]. Their primary role is that of a ‘bone phagocyte’. They are responsible for debridement, resorption of the cartilaginous callus, resorption of the bony callus, resorbing the tunnels required for vasculature and nerves, and together with osteoblasts balance bone remodeling [25]. Upon activation, osteoclasts adhere to the bone surface, form a ruffled border, and create a tight seal with the mineralized surface termed a resorption pit [52]. This sealed compartment is then acidified by pumping in hydrogen ions to dissolve the hydroxyapatite crystal and lysosomal enzymes are secreted into the resorption pit to digest the proteinaceous material.

Recently, studies have further elucidated the immune cell signaling that regulates osteoclast activation. During fracture healing, monocytes are recruited to the site and differentiate to osteoclasts. Osteoclasts, which reside on mineralized bone surfaces, are primarily activated by receptor activator of nuclear factor kappa-B ligand (RANKL) binding to the osteoclast cell surface receptor RANK. Osteoblasts appear to be the primary source of RANKL during homeostasis and fracture healing; however, NK cells and activated T cells are also able to produce RANKL during fracture healing. Conversely, osteoprotegerin (OPG) is a decoy receptor that binds to RANK and inhibits RANKL binding, thereby preventing osteoclast activation. OPG is secreted by osteoblasts during homeostasis and fracture healing and by B cells during fracture healing.

This inflammatory signaling combines with a resorption-based signaling to create the communication mechanism that regulates bone remodeling. As osteoclasts resorb the bone matrix, proteins such as bone sialoprotein and osteopontin, which are intercalated within the mineralized matrix and bound to collagen and hydroxyapatite, are freed and able to signal to local osteoblasts as the RGD motif of these proteins binds to the α_v_β_3_ cell surface receptor [53, 54].

### T-Lymphocytes and B-Lymphocytes

T lymphocytes and B lymphocytes (also known as T cells and B cells) are hematopoietic cells of the lymphoid lineage. They constitute the two cell types of adaptive immunity and while their lineage can be further classified and subdivided, for the purpose of this review they will simply be classified as T cells and B cells. Depletion of T cells or of B cells leads to diminished bone health and decreased fracture healing [55, 56]. Mice lacking T cells and B cells have been shown to have stiffer bones that are more susceptible to fracture [57].

T cells and B cells seem to have cell-signaling roles near the end of the inflammatory phase and again during the mineralization phase [58]. During later stages of the inflammatory phase, T cells produce RANKL to recruit, differentiate, and activate osteoclasts; likely in an effort to remove the fibrin thrombus in preparation of the cartilaginous callus. During this time, B cells are involved in suppression of the pro-inflammatory signals IFN-γ, TNF-α, and IL-2 [59]. Concurrently, B cells produce OPG, thereby regulating osteoclastic differentiation and activity [60–62].

Recent findings have placed more attention on the role of the cytokine IL-17 secreted by T cells. IL-17 has been shown to be an immunomodulator, able to induce anti-inflammatory functions from mesenchymal stromal cells [63] and has been shown to induce osteogenic differentiation and activity, aiding in osteoblast maturation [56]. Furthermore, IL-17 plays a role during the remodeling phase of the fracture callus as it increases expression and secretion of RANKL, leading to enhanced proliferation and activation of osteoclasts [64].

### Natural Killer Cells

Natural killer cells (NK cells) are hematopoietic cells of the lymphoid lineage. The immunological function of NK cells is to recognize foreign or virally infected cells and induce apoptosis or cell lysis through cytotoxic granules [65]. Little is known about the function of natural killer cells in fracture healing. It is possible that NK cells play a role in removal of damaged cells located at the site of injury; however, conditions at the fracture site have been shown to inhibit NK cell-based cell lysis [66]. It is more likely that NK cells play a signaling role in debridement of the injured tissue recruiting inflammatory cells and osteoclasts as they are known to produce IFN-γ and RANKL [67]. NK cells may also play a role in tissue deposition through recruitment of mesenchymal progenitor cells at a later stage of fracture repair as they secrete CXCL7 [68].

## Mesenchymal Signals to Immune Cells

The term “licensing” has been coined to describe the anti-inflammatory response of mesenchymal stromal cells (MSCs) to inflammatory cytokines secreted by immune cells [69]. IL-17 secreted by T-lymphocytes induces an iNOS-based immunosuppressive response in MSCs [63]. Secretion of TNF-α by immune cells has been shown to induce anti-inflammatory activity of MSC population via activation of NF-κB [70]. Using models of graft versus host disease, INF-γ was identified in licensing MSCs to help suppress the immune response [71]. Interestingly, IL-1α or IL-1β alone are not able to elicit a licensing response from MSCs but do so in the presence of INF-γ [72]. Novel interactions and the effects of inflammatory molecules on mesenchymal cells continue to be discovered. As this phenomenon is studied, the information gained may help to shed light on processes involved in bone fracture healing and potential therapeutic interventions.

## Immune Dysfunction and Fracture Healing

In the clinical setting, the importance of healthy immune function on fracture healing is clear. Neither a muted nor an elevated immune response is advantageous during bone fracture healing. HIV-positive patients display slower bone fracture healing, increased occurrence of fragility fractures, and increased risk of developing osteoporosis [73]. Conversely, in conditions of autoimmune disease, such as lupus or rheumatoid arthritis, bone fracture healing is also inhibited [74, 75]. With chronic inflammation, as in diabetes, obesity, or aging, inflammatory signaling and processing are dysregulated, leading to a chronic state of elevated inflammation, which is associated with poor fracture healing [37]. For example, in diabetes, increased levels of TNF-α lead to increased apoptosis of chondrocytes and premature resorption of the cartilaginous callus [76, 77].

## Inflammatory Cells as a Target for Treatment?

In aged patients, fracture healing occurs at a slower pace and has a higher occurrence of non-union than in young patients. In conditions of metabolic dysregulation, fracture repair is likewise hindered and often results in bone that is weaker than the original tissue [78]. This lack of structural integrity leads to a higher rate of re-fracture and a higher rate of revision surgery in implants. The information gained from investigating osteoimmunology in fracture healing could lead to novel treatment strategies and a better prognosis for bone injury patients.

Scaffolds made of various biomaterials have been employed in the surgical management of non-healing fractures. These scaffolds provide the structural template for tissue regeneration. Historically, these scaffolds were designed to be inert to minimize the host’s immune response to the foreign body. However, these scaffolds often lead to activation of the innate and adaptive immune system [79]. Since then, our understanding of the immune response in wound healing has vastly improved, and there has been a growing interest in developing scaffolds with immune-modulating capacities. Recent studies have shown that various biomaterials can affect in vivo macrophage function, altering the polarization of macrophages [80–82]. Most of these studies have been performed on animal models. It remains to be seen whether these scaffolds will elicit similar effects in humans.

Efforts to modulate the immune system to improve fracture repair has been documented in the literature. Platelet-rich plasma (PRP) therapy is an autologous blood product in which patient’s own platelets have been concentrated, and injected locally at the site of injury [83–86]. Although there are some studies suggesting improvement in fracture healing with PRP therapy in humans, the sample size of these studies were small and only a few were randomized controlled studies. While platelets may play a role in the initial inflammatory cascade, the mechanism by which concentrated platelets promote fracture healing is largely unknown.

## Conclusion

Immune cells play a critical role in bone fracture healing. These cells serve as the initial responders at the site of injury, mending vasculature, and initiating cascades of signals to recruit cells to carry out the repair processes. Osteoimmunity is a developing research field and more work must be done to better appreciate the biological significance of immune cells in bone regeneration.
